# LINC00461 SNPs rs933647 and rs201864123 modify the risk of adenoid hypertrophy susceptibility for children in South China

**DOI:** 10.3389/fgene.2025.1509053

**Published:** 2025-02-21

**Authors:** Chao Hou, Xilian Luo, Xin Wan, Kaining Chen, Zhongren Xian, Kaixiong Xu, Yingjia Zeng, Chenlu Wang, Wan Yang, Zilin Zheng, Yueling Lin, Zhaojin Lu, Yanqiu Chen, Di Che, Xiaoqiong Gu

**Affiliations:** ^1^ Department of Otolaryngology, Guangzhou Women and Children’s Medical Center, Guangzhou Medical University, Guangzhou, Guangdong, China; ^2^ Guangdong Provincial Key Laboratory of Research in Structural Birth Defect Disease, Department of Clinical Biological Resource Bank, Guangzhou Women and Children’s Medical Center, Guangzhou Institute of Pediatrics, Guangzhou Medical University, Guangzhou, China; ^3^ Medicine Inspection Department of the Fifth Affiliated Hospital of Guangzhou Medical University, Guangzhou, Guangdong, China; ^4^ Department of Laboratory Medicine, The Second Affiliated Hospital, School of Medicine, South China University of Technology, Guangzhou, China; ^5^ School of Clinical Medicine, Kunming Medical University, Kunming, Yunnan, China

**Keywords:** adenoidal hypertrophy, susceptibility, LINC00461, microRNA, SNP

## Abstract

**Background:**

Adenoidal hypertrophy (AH) is commonly observed in childhood and closely linked to obstructive sleep apnea (OSA). Despite the high prevalence of AH, its pathophysiological mechanisms remain incompletely understood. We attempt to explore this issue from a genetic perspective. Elevated levels of LINC00461 have been identified in OSA tissues. We aimed to explore the relationship between susceptibility to adenoid hypertrophy and LINC00461 gene polymorphisms.

**Methods:**

We genotyped the LINC00461 single nucleotide polymorphisms (SNPs) rs933647 and rs201864123 in 546 AH patients and 574 healthy controls. Odds ratios (ORs) and 95% confidence intervals (CIs) were calculated to assess the association between the SNPs and AH risk. The SIPI (Susceptible-Infected-Protected-Infected) method was utilized to analyze SNP-SNP interactions between rs933647 and rs201864123.

**Results:**

Our study found that the rs933647 GA polymorphism was associated with an increased risk of AH. Similarly, the T allele of SNP rs201864123 increased AH risk in southern Chinese children. Furthermore, SIPI analysis demonstrated an interaction between these SNPs associated with adenoid hypertrophy risk.

**Conclusion:**

The LINC00461 rs933647 GA genotype and rs201864123 T variant may contribute to the susceptibility of AH in the child population of China.

## Introduction

Adenoids, also referred to as pharyngeal tonsils, are essential components of the Waldeyer’s lymphatic ring complex (Arambula et al., 2021). Adenoid hyperplasia typically shows robust progression between ages 2 and 6, with atrophy generally beginning around age 10 (Gill et al., 2021). According to a recent meta-analysis, the prevalence of adenoid hypertrophy in a randomly selected representative sample of children and adolescents was determined to be 34.46% (Pereira et al., 2018). Key symptoms associated with adenoid hypertrophy include nasal congestion, sleep apnea, snoring, and the characteristic “adenoid face,” resulting from chronic airway obstruction (Atilla et al., 2018). Additionally, adenoid hypertrophy impacts childhood cardiovascular health (Kontos et al., 2020), mouth breathing (Milanesi et al., 2018), and increased risks of periodontal disease and dental caries (İnönü-Sakallı et al., 2021). The conventional approach to managing adenoidal hypertrophy typically involves adenoidectomy, which has become one of the predominant surgical interventions in pediatric practice (Johnston et al., 2017). Adenoid tonsillectomy is the largest number of otolaryngology surgeries (González Poggioli et al., 2008). However, a substantial number of adenoidectomy procedures impose a significant socioeconomic burden (Curtis et al., 2015).

Despite its prevalence, the precise pathophysiology of adenoid hypertrophy remains incompletely understood (Atilla et al., 2018). Currently, it is widely accepted that adenoid hypertrophy can result from various etiological factors, encompassing both infectious and non-infectious origins. Among infectious causes, adenoid hypertrophy can be attributed to viral and bacterial pathogens (Chorney and Zur, 2021). Non-infectious etiologies include gastroesophageal reflux (Niu et al., 2018), allergic rhinitis, and exposure to smoke, among others (Evcimik et al., 2015). Additionally, there is suggestion that adenoid hypertrophy may be associated with heredity (Friberg et al., 2009), and potentially linked to lymphoma and nasal malignancies (Rout et al., 2013).

The pathogenesis of adenoid hypertrophy presents a significant challenge for pediatricians, particularly in identifying reliable biomarkers for early diagnosis and prognosis, which could inform personalized treatment strategies. The identification of accurate and dependable biomarkers remains a key issue in assessing prognosis and guiding personalized treatment approaches. This challenge has piqued our interest in exploring novel markers associated with adenoid hypertrophy.

## Materials and methods

### Sample selection

The present investigation received approval from the institutional review board of Guangzhou Women and Children’s Medical Center, Guangzhou Medical University (2022285B00). A total of 546 patients diagnosed with adenoid hypertrophy and 574 healthy individuals from Guangzhou Women and Children’s Medical Center, Guangzhou Medical University were recruited for this study. Written consent was obtained from all participants upon their enrollment. The diagnostic criteria for adenoid hypertrophy were established based on the Sleep Medicine Technical Standards Approval Committee of the World Federation of Chinese Medicine Societies and are as follows: (1) Fiberoptic nasopharyngoscopy revealed 76%–100% of nostrils after Adenoid obstruction; (2) The clinical manifestations are snoring, nasal congestion and mouth opening breathing; (3) The imaging examination showed that the ratio of the vertical distance from the most prominent point of the adenoid (A) to the lateral surface of the occipital clivus skull to the width of the nasopharynx cavity at the most prominent part of the adenoid (N) A/N > 0.6. Adenoid hypertrophy was graded in accordance with Parikh’s classification: Grade 1: indicated adenoid tissue not in contact with the adjacent structures; Grade 2: indicated adenoid tissue in contact with the torus tubarius; Grade 3: indicated adenoid tissue in contact with the vomer; and Grade 4: indicated adenoid tissue in contact with the soft palate (Parikh et al., 2006).

### Polymorphism selection

LINC00461 gene SNPs with potential functions were retrieved from the dbSNP database (http://www.ncbi.nlm.nih.gov/) and SNPinfo software (http://snpinfo.niehs.nih.gov/). The selection criteria were summarized as follows: (1) SNPs retrieval was based on NCBI recommended transcript sequences as a reference. The minor allele frequency (MAF) was >0.05 for Eastern Asia subjects reported in 1,000 Genomes (https://www.ncbi.nlm.nih.gov/variation/tools/1000genomes/); (2) at least one potential regulation function such as roadmap epigenomics, transcript factor binding site, CpG islands. (3) SNPs in low linkage disequilibrium (LD) with each other (*r* ^2^ < 0.2). Two SNPs (rs933647 G>A and rs201864123 T>G) were screened out for analysis.

### DNA extraction and SNP genotyping

The extraction of genomic DNA from peripheral blood was performed using the standard procedure with the QIAamp DNA Blood Mini Kit (QIAGEN, Valencia, CA). The genotyping of the SNPs was carried out utilizing the TaqMan SNP genotyping assay. Laboratory technicians were blinded to the sample information and duplicate aliquots identification to ensure unbiased analysis. To ensure quality control, repetitive SNP genotyping was conducted on a randomly selected 10% of the samples from both the cases and controls. Notably, all SNPs displayed a genotype concordance rate of 100%.

### Statistical analysis

The chi-square (χ^2^) test was employed to compare the variations in clinical variables and genotype frequencies of LINC00461 rs933647/rs201864123 between patients with adenoid hypertrophy and healthy controls. Additionally, multivariate logistic regression analysis was performed to calculate the odds ratio (OR) and, 95% confidence interval (CI) for the risk of adenoid hypertrophy, stratified by age and gender. All of the aforementioned statistical analyses were conducted utilizing SAS software (Version 9.3, SAS Institute, United States). A significance level of *P*<0.05 was considered statistically significant.

### SNP interaction pattern identifier (SIPI)

The SIPI detects 45 interaction models, encompassing both original and reverse coding for inheritance mode and risk category grouping to analyze the model structure. The selection of the optimal interaction pattern is determined by the Bayesian Information Criterion (BIC), which balances the model’s fit and complexity. The best model is identified based on the lowest BIC value among the 45 models. In terms of statistical significance, the SIPI and SNPassoc consider a P-value threshold of <0.001 (equivalent to 0.05/45) and <0.01 (equivalent to 0.05/5) respectively, with the Bonferroni correction applied (Lin et al., 2017).

## Results

Our investigation into this area prompted us to explore potential markers linked to adenoid hypertrophy using collaborative efforts and diverse datasets from the GEO database (Supplementary Tables S1, S2). In our analysis, we discovered a correlation between and LINC00461 can regulate miR-342-3p, using Lncbase prediction by DIANA Tools (https://diana.e-ce.uth.gr/lncbasev3/interactions). Furthermore, bioinformatics analysis (Supplementary Figures S1A, B) and functional predictions (Supplementary Figures S1C–E) indicated an association between miR-342-3p and OSA. Notably, adenoid hypertrophy (AH) stands as the most prevalent risk factor contributing to the development of OSA (Friberg et al., 2009).

Our team screened two OSAHS-associated non-coding group gene expression array datasets (Supplementary Table S1) and five OSAHS-associated transcriptome gene expression array datasets (Supplementary Table S2) from the GEO database in the preliminary study, and used bioinformatics methods to perform differential gene analysis (Supplementary Table S3). We obtained two related miRNAs (Supplementary Figures S1B). miR-142-5p and miR-342-3p were significantly downregulated in both soft palate muscle and tonsil tissues of OSAHS patients by database analysis. To improve the accuracy of the screening, we included genes that met the threshold in at least three datasets as candidate differential genes, and a cluster containing 46 transcriptome differential genes was obtained by intersection analysis. In this study, we explored which genes miR-142-5p and miR-342-3p might affect the biological process of OSAHS by targeting them through TargetScan, ENCORI, miRDB, and miRtarbase platform predictions, and combined with the OSAHS-associated transcriptome differential gene clusters obtained in the previous stage, we found that: miR-142-5p could target RB1CC1, RPS6KA5, and ZBTB20 in the cluster, while miR-342-3p could target MTDH in the cluster, which was suggested by GO analysis (Gene Ontology) annotation that these four target genes might be related to the biological processes of inflammation and cell proliferation in OSAHS (Supplementary Table S4). It is noteworthy that MTDH is considered to be closely associated with tissue inflammation (Wang et al., 2021).

We included 546 children diagnosed with adenoid hypertrophy and 574 children who attended our hospital for health examinations without a history of adenoid hypertrophy as control subjects. As shown in Table 1, there were no statistically significant differences in age (P = 0.370) and gender (P = 0.623) between the two groups.

**TABLE 1:** Characteristics in adenoidal hypertrophy patients and healthy controls.

Variables	AH	Controls	P^a^
Total	546	574	
Age range (month)	12–160	1–158	
Mean ± SD	59.6 ± 24.55	38.92 ± 38.72	
≤120	548	527	0.370
>120	26	19	
Gender			
Male	353	363	0.623
Female	193	211	

^a^ Two-sided χ2 test for distributions between healthy controls and KD patients.

### Association between LINC00461/rs933647 polymorphisms and adenoid hypertrophy

The genotype frequency distribution of the LINC00461/rs933647 polymorphism in the adenoid hypertrophy and control groups is detailed in Table 2 χ^2^ tests revealed significant differences in the rs933647 SNP between the control and adenoid hypertrophy groups (*P* < 0.05). In unadjusted analysis, the LINC00461/rs933647 GA genotype was associated with adenoid hypertrophy (*OR* = 1.62, 95% *CI* = 1.24–2.13), and this association remained significant after adjusting for age and gender (adjusted *OR* = 1.67, 95% *CI* = 1.26–2.23). Specifically, the risk of adenoid hypertrophy was higher in individuals with the GA genotype compared to those with GG in multiple regression analysis (*OR* = 1.62, 95% *CI* = 1.24–2.13), and after adjustment for sex and age, the *OR* was 1.67, with a 95% *CI* of 1.26–2.23. In the additive model, the *OR* was 1.40 with a 95% *CI* of 1.09–1.78, and after adjustment for sex and age, the *OR* was 1.40 with a 95% *CI* of 1.09–1.78. Similarly, in the dominant model, the *OR* was 1.56 with a 95% *CI* of 1.20–1.93, and the adjusted *OR* was 1.58 with a 95% *CI* of 1.20–2.08. Both the additive model (adjusted *OR* = 1.40, 95% *CI* = 1.09–1.78) and the dominant model (adjusted *OR* = 1.58, 95% *CI* = 1.20–2.08) suggest that this polymorphism is a risk factor for adenoid hypertrophy.

**TABLE 2:** Genotype Frequency distribution of polymorphisms in LINC00461/rs933647 between AH cases with healthy control.

Genotype	AH cases (n = 546, %)	Healthy control (n = 574, %)	P-valuea	Or (95% CI)	P-value	Adjusted OR (95%CI)	Adjusted P-value
**GG**	**368 (67.40)**	**438 (76.31)**	**0.0021**	**1.00**		**1.00**	
**GA**	**165 (30.22)**	**121 (21.08)**		**1.62 (1.24–2.13)**	**0.0005**	**1.67 (1.26–2.23)**	**0.0004**
**AA**	**13 (2.38)**	**15 (2.16)**		1.03 (0.49–2.20)	0.9358	0.85 (0.37–1.93)	0.6902
**Additive**				**1.40 (1.11–1.76)**	**0.0048**	**1.40 (1.09–1.78)**	**0.0078**
**Dominant**	**178 (32.60)**	**136 (23.24)**	**0.0009**	**1.56 (1.20–2.03)**	**0.0009**	**1.58 (1.20–2.08)**	**0.0012**
**Recessive**	533 (97.62)	559 (97.39)	0.8033	0.91 (0.43–1.93)	0.8039	0.74 (0.32–1.68)	0.4705

*Bold values indicate statistically significant results (P <0.05).

### Association between LINC00461/rs201864123 polymorphism and adenoid hypertrophy

The genotype frequency distribution of the LINC00461/rs201864123 polymorphism in the AH case and control groups is presented in Table 3 χ^2^ tests revealed significant differences in rs201864123 between the control and AH groups (*P* < 0.05). In multiple regression analysis, the risk of adenoid hypertrophy was higher for individuals with the GT genotype (adjusted *OR* = 4.71, 95% *CI* = 1.03–21.58) and TT genotype (adjusted *OR* = 6.03, 95% *CI* = 1.35–27.06) compared to those with GG genotype. In the additive model, after adjusting for relevant factors, the *OR* was 1.50 with a 95% *CI* of 1.10–1.94, indicating that each copy of the T allele increases the risk of AH. Similarly, in the dominant model, the adjusted *OR* was 5.76 with a 95% *CI* of 1.29–25.82, suggesting a significant association between carrying at least one T allele and AH risk. Additionally, the negative model (adjusted *OR* = 1.40, 95% *CI* = 1.02–1.91) also indicated a potential increase in AH risk associated with the T allele. These findings collectively suggest that the T allele of the LINC00461/rs201864123 polymorphism is associated with an increased risk of adenoid hypertrophy in southern Chinese children.

**TABLE 3:** Genotype Frequency distribution of polymorphisms in LINC00461/rs201864123 between AH cases with healthy control.

Genotype	AH cases (n = 546, %)	Healthy control (n = 574, %)	P-valuea	Or (95%CI)	P-value	Adjusted OR (95% CI)	Adjusted P-value
GG	2 (0.37)	16 (2.79)	0.0009	1.00		1.00	
GT	92 (16.85)	114 (19.86)		6.46 (1.45–28.80)	0.0145	4.71 (1.03–21.58)	0.0460
TT	452 (82.78)	444 (77.35)		8.14(1.86–35.62)	0.0053	6.03 (1.35–27.06)	0.0189
Additive				1.49 (1.14–1.95)	0.0037	1.50 (1.10–1.94)	0.0097
Dominant	544(99.63)	558(97.21)	0.0006	7.80 (1.79–34.08)	0.0063	5.76 (1.29–25.82)	0.0220
Recessive	94(17.22)	130(22.65)	0.0228	1.41 (1.05–1.89)	0.0235	1.40 (1.02–1.91)	0.0347

### SNP–SNP interactions

In Table 4, the combination of rs933647 GA/AA+ rs201864123 GT/TT genotypes significantly increased the risk of adenoid hypertrophy compared to the rs933647 GG + rs201864123 GG genotypes (adjusted OR = 11.61, 95% CI = 1.44–93.37). Furthermore, the presence of rs933647 GA/AA+ rs201864123 GT/TT genotypes was independently associated with a 1.63-fold increase in the risk of adenoid hypertrophy (adjusted OR = 1.63, 95% CI = 1.23–2.15). Using the SIPI method, we analyzed the interaction between the SNP polymorphisms of rs933647 and rs201864123, revealing a significant interaction with adenoid hypertrophy (Wald_Chisq = 12.58, *P* = 0.0004). These results indicate that there is a synergistic effect between the rs933647 and rs201864123 polymorphisms in influencing the risk of adenoid hypertrophy, suggesting a potential combined genetic susceptibility in southern Chinese children.

**TABLE 4:** Genotype Frequency distribution of polymorphisms in LINC00461/rs933647 and rs201864123 between AH cases with healthy control.

Genotype	AH cases (n = 546, %)	Healthy control (n = 574, %)	P-valuea	Or (95% CI)	P-value	Adjusted OR (95% CI)	Adjusted P-value
**A**	**1 (0.18)**	**11 (1.92)**	**<0.0001**	**1.00**		**1.00**	
**B**	**368 (67.40)**	**432 (75.26)**		**9.37 (1.20–72.92)**	**0.0326**	7.28 (1.07–3.56)	0.0610
**C**	**177 (32.42)**	**131 (22.82)**		**14.86 (1.90–116.55)**	**0.0102**	**11.61 (1.44–93.37)**	**0.0212**
**Trend**			**0.0003**	**1.70 (1.32–2.95)**	**<0.0001**	**1.69 (1.29–2.21)**	**0.0001**
**A + B**	**369 (67.58)**	**443 (77.18)**		**1.00**		**1.00**	
**C**	**177 (32.42)**	**131 (22.82)**		**1.62 (1.25–2.11)**	**0.0003**	**1.63 (1.23–2.15)**	**0.0006**

Var1 Var2		Model		BIC Wald_Chisq		Wald_p	
**SNP1 SNP2**		**DR_M2_int_r2**		**12.58**		**0.0004**	

Genotype A = rs933647 GG + rs201864123 GG.

Genotype B = rs933647 GA/AA + rs201864123 GG, and rs933647 GG + rs201864123 GT/TT.

Genotype C = rs933647 GA/AA + rs201864123 GT/TT.

*Bold values indicate statistically significant results (P <0.05).

## Discussion

The pathophysiological mechanism underlying adenoid hypertrophy remains uncertain. However, several studies have suggested a potential correlation between adenoid hypertrophy and gene polymorphisms. For example, research on SNPs in the Ugrp2 gene has shown that mutations in these SNPs increase the risk of developing adenoid hypertrophy (Atilla et al., 2018). In this study, the Ugrp2 (IVS1-89T>G) TG and (c.201delC) CdelC genotypes and their minor alleles were associated with a considerable increase in the risk of adenoid hypertrophy compared with the controls. Regarding IL-10 rs1800896, the GG genotype may confer resistance against adenoid hypertrophy and restrict adenoid tissue growth (Lomaeva et al., 2022). Babademez et al. (2016) reported that TLR4 gene polymorphisms predispose individuals to adenoid hypertrophy. Additionally, Grasso et al. investigated Italian children with adenotonsillar hypertrophy, focusing on genetic variations in the MBL2 gene, and found that individuals with the MBL2 00 genotype might serve as a prognostic marker for those affected (Grasso et al., 2007). Numerous studies have confirmed the association between SNPs and susceptibility to adenotonsillar hypertrophy; however, there is currently no research on the relationship between SNPs in long non-coding RNAs and adenotonsillar hypertrophy. Our study revealed an association between adenoid hypertrophy and gene variants in LINC00461, identifying the LINC00461/rs933647 GA genotype and the LINC00461/rs201864123 TT and GT genotypes as risk factors. This marks the first study to uncover such links, suggesting that LINC00461 gene variants may influence adenoid hypertrophy susceptibility in southern China.

Long non-coding RNAs (lncRNAs) are RNA molecules longer than 200 nucleotides that do not encode proteins (Djebali et al., 2012). These lncRNAs can modulate gene expression through epigenetic, transcriptional, and posttranscriptional mechanisms. Many human diseases have been shown to be associated with deregulated lncRNAs, rendering them potential therapeutic targets and biomarkers for differential diagnosis (Pokorná et al., 2024). Additionally, single nucleotide variations, known as SNPs, have the potential to alter the function of lncRNAs and influence susceptibility to various diseases (Lu et al., 2022). Among the 7,666 intergenic lncRNAs examined, LINC00461 showed the highest conservation, indicating a significant evolutionary role for this particular lncRNA (Deguchi et al., 2017). Based on current research on LINC00461, we hypothesize that it may play a potential role in the pathophysiological mechanisms of adenotonsillar hypertrophy. A study on Major Depressive Disorder and Atopic Diseases found that polymorphisms in LINC00461 are associated with atopic diseases (Cao et al., 2021). Research has confirmed a strong correlation between atopic diseases and adenotonsillar hypertrophy (Modrzyński and Zawisza, 2003). First, LINC00461 is associated with tissue inflammation. Overexpression of LINC00461 was found to elevate the expression of IL-10, an anti-inflammatory factor, in chondrocytes (Zhang et al., 2020). A comprehensive study investigating the association between major depressive disorder and atopic diseases identified a significant correlation involving LINC00461 and both conditions (Cao et al., 2021), underscoring its role in inflammation and tissue dysplasia. Some studies have confirmed that LINC00461 exhibits significantly higher expression levels in nasal tissues compared to bronchial tissues. (Giovannini-Chami et al., 2018). We infer that the role of LINC00461 is related to inflammation in the nasal cavity. Furthermore, LINC00461 may be involved in tissue hyperplasia. Overexpression of LINC00461 has demonstrated a significant promotion of cell proliferation *in vitro* in tests involving colorectal cancer cells (Yu et al., 2019). Increased expression of LINC00461 has been linked to enhanced chondrocyte proliferation, cell cycle progression, inflammation, and extracellular matrix degradation. So, based on these observations, the heightened expression of LINC00461 in nasal tissues suggests a potential association with nasal inflammation, which could potentially lead to the proliferation of adenoid tissue and consequently contribute to adenoid hypertrophy. It is plausible that these SNPs could induce alterations in the inflammatory pathways, thereby influencing the pathophysiology of adenoid hypertrophy.

Abnormal expression of LINC00461 is closely associated with the clinical pathological features of diseases, possibly because lncRNAs can regulate various miRNAs. (Zhang et al., 2022). Some studies have demonstrated that lncRNAs are involved in multiple biological processes including oncogenic transformation by regulating gene expression (Deguchi et al., 2017). They affect the chromatin structures and RNA interactions, such as those with the microRNA (miRNA) sponge, which upregulates protein expression by inhibiting miRNA binding to their targets (Schmitt and Chang, 2016). Studies showed that abnormal expression of long intergenic noncoding RNA deregulates signaling pathways due to the disrupted free microRNA pool. Impaired signaling pathways lead to abnormal cell proliferation and inhibit cell death, thereby contributing to the development of diseases. Our study, based on the DIANA database and findings by Karginov FV et al. (Karginov and Hannon, 2013), reveals a regulatory link between LINC00461 and miR-342-3p. Analysis of two GEO datasets (GSE135917, GSE49800) from OSA patient tissues shows upregulation of LINC00461, with significant differences, further supporting the disease-regulatory interaction (Supplementary Figure S1). AH is characterized by a localized inflammatory response causing glandular enlargement. miR-342-3p has been implicated in inflammatory processes in various diseases, including respiratory syncytial virus infection (Wang et al., 2017) and macrophage inflammation regulation (Wang et al., 2019). It also affects nasopharyngeal carcinoma (NPC) progression, with downregulation linked to poor prognosis and inhibition of growth and invasion via FOXQ1 repression and Cdc42 pathway targeting (Cui and Zhao, 2019). Moreover, miR-342-3p inhibits NPC tumor growth and invasion by directly targeting the Cdc42 pathway (Shi et al., 2018). TP73-AS1 also promotes NPC progression by regulating miR-342-3p and macrophage communication via exosomes (Yao et al., 2022). Thus, miR-342-3p plays a pivotal role in inflammation and tumor progression. Given the potential role of miR-342-3p in adenoid hypertrophy, we hypothesize that LINC00461’s high expression in the nasal cavity induces inflammation via miR-342-3p, leading to adenoid hyperplasia and hypertrophy.

In our present study, we identified the rs933647 GA/AA and rs201864123 GT/TT genotypes as risk factors for adenoid hypertrophy. Moreover, recent research suggests the presence of interplay between these SNPs. In a study examining SCGB1D4 gene polymorphisms and adenoid hypertrophy, it was noted that all analyzed SNPs exhibited synergistic effects with each other (Özdaş et al., 2017). Using SNP interaction pattern identification tools, we analyzed the interaction between rs933647 and rs201864123 SNPs and discovered a significant association with adenoid hypertrophy (*P* < 0.001). Lin et al. (Lin et al., 2023) suggest that interactions between SNPs in folate metabolism-related genes have a more profound impact on tumor aggressiveness in patients with certain genotypes. Wagner et al. (Wagner et al., 2020) found an SNP-SNP interaction between rs10815225 and rs7421861 associated with risk of clear cell renal cell carcinoma. Within our study, compared to the rs933647 GG + rs201864123 TT combination, other SNP combinations posed a higher risk for adenoid hypertrophy. Additionally, the odds ratio associated with this combined effect exceeded that of individual SNPs, underscoring a notable synergistic effect between the two aforementioned SNPs.

The genetic polymorphism of LINC00461 offers a promising new biomarker for the early identification of adenoid hypertrophy. As a long non-coding RNA, LINC00461 has been recognized as a potential diagnostic marker in other diseases (Xu et al., 2022). In a study on osteoarthritis, LINC00461 was proposed as a potential target for both diagnosis and therapy (Zhang et al., 2020). However, the development of adenoid hypertrophy is influenced not only by genetic factors but also by environmental factors, such as infections and allergies (Chorney and Zur, 2021). Therefore, while genetic screening tools show promise, they should be combined with clinical symptoms and other biomarkers to improve diagnostic accuracy. Screening for SNPs associated with adenoid hypertrophy could provide valuable insights for early diagnosis, particularly in patients with atypical clinical presentations. Moreover, genetic testing based on SNPs may be integrated with existing clinical evaluation methods to enhance diagnostic precision. Nevertheless, the role of the LINC00461 gene in adenoid hypertrophy is not yet fully understood, underscoring the need for further functional studies to explore how polymorphisms within the LINC00461 gene influence the risk of adenoid hypertrophy by modulating immune responses, cell proliferation, or other biological processes. To further validate the findings, multicenter studies should be conducted to assess the stability and association of LINC00461 SNPs across diverse populations. This will help ensure the general applicability of the results and provide a more robust foundation for clinical applications.

However, it is crucial to acknowledge the limitations of this inaugural study on LINC00461 genetic polymorphisms in Chinese children with adenoid hypertrophy. Firstly, the study included only 546 patients diagnosed with adenoid hypertrophy and 574 controls, which may affect statistical power due to the relatively small sample size. Secondly, our investigation focused solely on rs933647 and rs201864123, leaving other potentially functional LINC00461 gene polymorphisms unexplored. Thirdly, logistic regression analysis adjusted only for gender and age, overlooking other potential predisposing factors for adenoid hypertrophy, such as childhood allergic diseases. Lastly, although we have established a connection between LINC00461 SNPs and adenoid hypertrophy, the exact role of LINC00461 in the pathogenesis of adenoid hypertrophy remains elusive. Consequently, additional functional studies are required to elucidate how LINC00461 gene polymorphisms may influence the risk of adenoid hypertrophy by modulating immune responses, cellular proliferation, or other underlying biological mechanisms.

## Conclusion

In conclusion, our study has established a significant correlation between SNPs in LINC00461 and adenoid hypertrophy among children from southern China. Our findings underscore that interactions among these SNPs are linked to an increased susceptibility to adenoid hypertrophy. However, it is important to recognize that SNPs in LINC00461 may represent association signals rather than definitive causative variants. Therefore, conducting functional studies to elucidate how individual SNPs in this genomic region influence the expression of LINC00461 is crucial. Furthermore, to strengthen our results, future investigations should involve larger sample sizes and mechanistic experiments. These efforts will enhance our understanding of the biological mechanisms underlying the association between LINC00461 SNPs and adenoid hypertrophy, potentially paving the way for targeted therapeutic strategies or preventive measures.

## Data Availability

We have published our original SNP genotyping data on figshare, link: https://figshare.com/s/99721a7797437ae73bd9.
