# Supplementary material for: LINC00461 SNPs rs933647 and rs201864123 modify the risk of adenoid hypertrophy susceptibility for children in South China
Source: Front Genet. 2025 Feb 21;16:1509053. doi: 10.3389/fgene.2025.1509053 (PMC11885279; doi:10.3389/fgene.2025.1509053)
Supplement: Supplementary file 1 [file Supplementaryfile1.pdf]

**Table S1. OSAHS-associated miRNA expression**

| Dataset | Species | Type | Sample | Group |
|---|---|---|---|---|
| GSE99239 | Homo sapiens | MicroRNA | Soft palate muscle | OSAHS =4 vs. Normal = 4 |
| GSE82064 | Homo sapiens | MicroRNA | Tonsil | OPSCC=78 vs.Normal= 18 |

**Table S2. OSAHS-associated transcriptome gene expression array dataset**

| Dataset | Species | Type | Sample | Group |
|---|---|---|---|---|
| GSE135917 | Homo sapiens | mRNA | Fatty tissue | OSAHS =10 vs. Normal = 8 |
| GSE75097 | Homo sapiens | mRNA | PBMC | OSAHS with pre-treatment = 34 vs. OSAHS with post-treatment = 14 |
| GSE71356 | Homo sapiens | mRNA | PBMC | OSAHS =4 vs. Normal= 4 |
| GSE7224 | Homo sapiens | mRNA | Tonsil epithelia | OSAHS=6 vs. Normal=7 |
| GSE103412 | Homo sapiens | mRNA | Mucosa/Blood | Tonsillar squamous cell carcinoma =16vs. Normal= 18 |

Our team screened two OSAHS-associated non-coding group gene expression array datasets (Table S1) and five OSAHS-associated transcriptome gene expression array datasets (Table S2) from the GEO database in the preliminary study, and used bioinformatics methods to perform differential gene analysis. We obtained two related miRNAs (Figure S1 B). miR-142-5p and miR-342-3p were significantly downregulated in both soft palate muscle and tonsil tissues of OSAHS patients by database analysis. To improve the accuracy of the screening, we included genes that met the threshold in at least three datasets as candidate differential genes, and a cluster containing 46 transcriptome differential genes was obtained by intersection analysis. In this study, we explored which genes miR-142-5p and miR-342-3p might affect the biological process of OSAHS by targeting them through TargetScan, ENCORI, miRDB, and miRtarbase platform predictions, and combined with the OSAHS-associated transcriptome differential gene clusters obtained in the previous stage, we found that: miR-142-5p could target RB1CC1, RPS6KA5, and ZBTB20 in the cluster, while miR-342-3p could target MTDH in the cluster. RB1CC1, RPS6KA5 and ZBTB20 in the cluster, while miR-342-3p could target MTDH in the cluster, which was suggested by GO analysis (Gene Ontology) annotation that these four target genes might be related to the biological processes of inflammation and cell proliferation in OSAHS (Table S4).

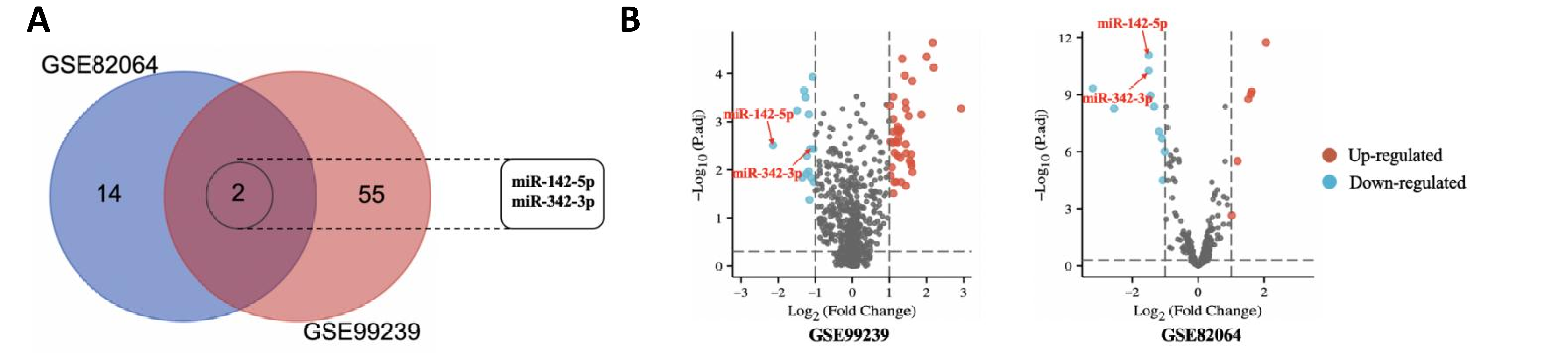


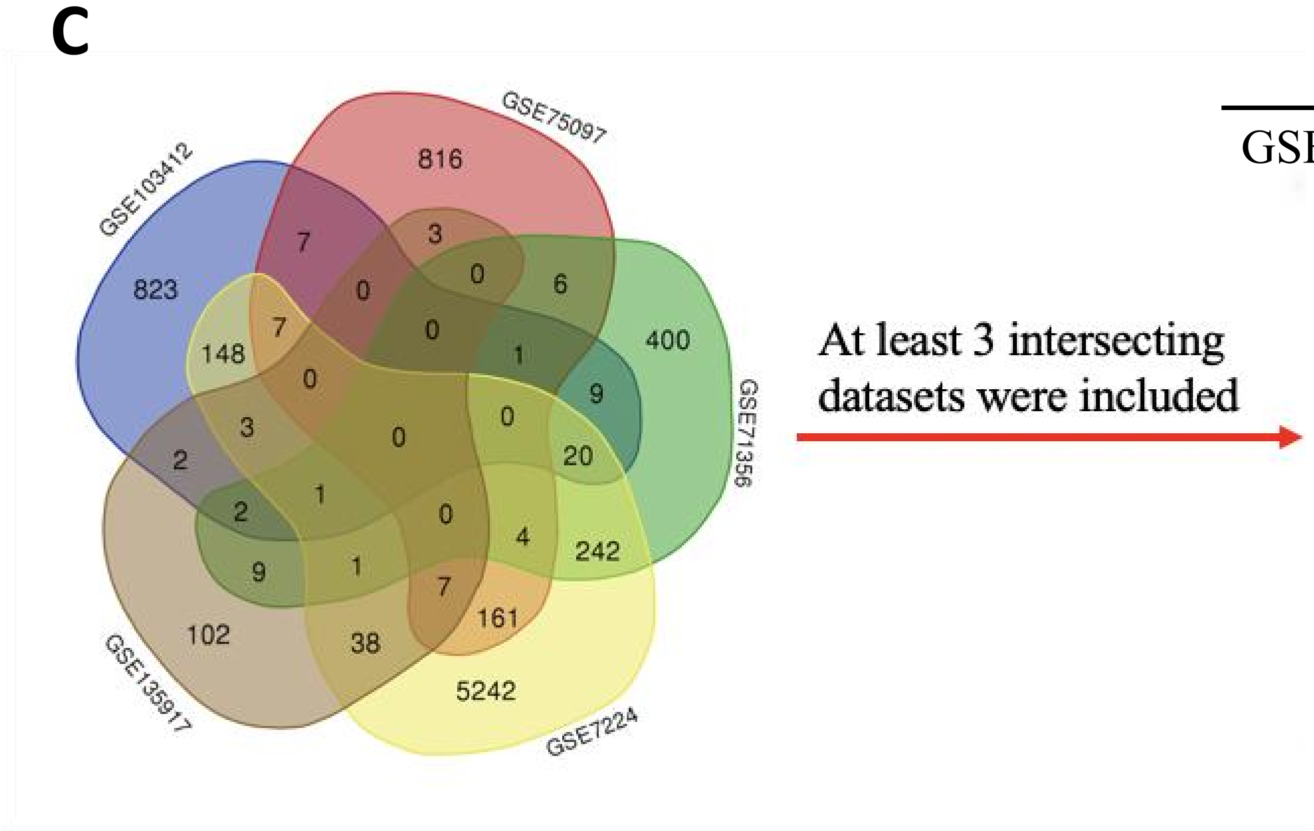


| Datasets | Intersecting gene numbers |
|---|---|
| GSE103412 GSE135917 GSE71356 GSE7224 | 1 |
| GSE103412 GSE71356 GSE75097 | 1 |
| GSE103412 GSE7224 GSE75097 | 7 |
| GSE103412 GSE71356 GSE7224 | 20 |
| GSE103412 GSE135917 GSE71356 | 2 |
| GSE103412 GSE135917 GSE7224 | 3 |
| GSE71356 GSE7224 GSE75097 | 4 |
| GSE135917 GSE7224 GSE75097 | 7 |
| GSE135917 GSE71356 GSE7224 | 1 |
| Total | 46 |

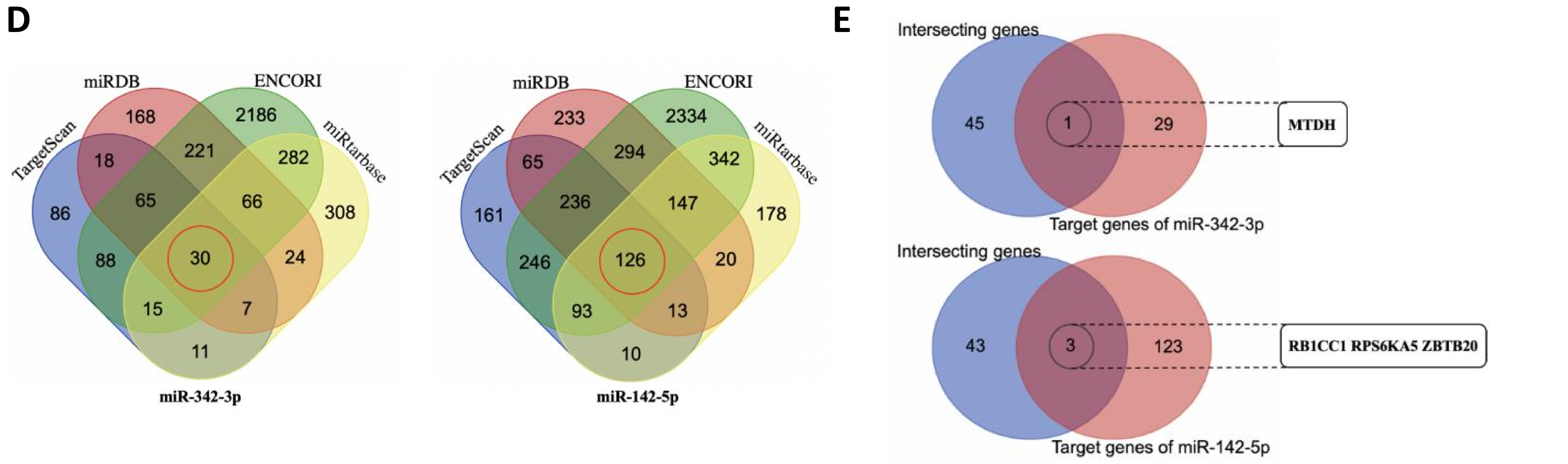


**Figure S1. The process of miR-342-3p prediction. (A)** Intersection analysis of OSAHS-associated miRNA gene datasets; **(B)** miR-142-5p and miR-342-3p are significantly downregulated in both soft palate muscle and tonsil tissues of OSAHS patients; **(C)** Intersection analysis of OSAHS-related transcriptomic gene datasets; **(D)** Intersecting miRNAs for multi-platform target gene prediction; **(E)** Intersection analysis of OSAHS-associated differential transcriptome genes with differential miRNA target genes based on the GEO platform.

**Table S3. Results of differential analysis of intersecting target genes**

| Dataset | MTDH | | RB1CC1 | | RPS6KA5 | | ZBTB20 | |
|---|---|---|---|---|---|---|---|---|
| | logFC | P.adj | logFC | P.adj | logFC | P.adj | logFC | P.adj |
| GSE135917 | / | / | 0.66 | 0.0175 | 0.59 | 0.0258 | 0.69 | 0.0149 |
| GSE75097 | 0.55 | 0.0241 | / | / | / | / | 0.53 | 0.0285 |
| GSE71356 | 0.81 | 0.0146 | / | / | 0.70 | 0.0163 | / | / |
| GSE7224 | / | / | 0.63 | 0.0469 | / | / | 1.57 | 0.0023 |
| GSE103412 | 0.63 | 0.0196 | 0.61 | 0.0201 | 0.59 | 0.0236 | / | / |

**Table S4. GO annotation of candidate genes with high relevance to OSAHS biological processes**

| Gene | GO ID | Qualified GO term |
|---|---|---|
| RB1CC1 | GO:0045793 | Involved in positive regulation of cell size |
| | GO:0008285 | Involved in negative regulation of cell population |
| | GO:0001934 | Involved in positive regulation of protein phosphorylation |
| | GO:2001237 | Involved in negative regulation of extrinsic apoptotic signaling pathway |
| RPS6KAS | GO:0006954 | Involved in inflammatory response |
| | GO:0051092 | Involved in positive regulation of NF-kappaB transcription factor activity |
| | GO:0070498 | Involved in interlcukin-1-mediated signaling pathway |
| | GO:0007173 | Involved in epidermal growth factor receptor signaling pathway |
| ZBTB20 | GO:0032755 | Involved in positive regulation of interleukin-6 production |
| | GO:0032760 | Involved in positive regulation of tumor necrosis factor production |
| | GO:0046889 | Involved in positive regulation of lipid biosynthetic process |
| | GO:0071333 | Involved in cellular response to glucose stimulus |
| MTDH | GO:0043123 | Involved in positive regulation of I-kappaB kinase/NF-kappaB signaling |
| | GO:0045766 | Involved in positive regulation of angiogenesis |
| | GO:0051092 | Involved in positive regulation of NF-kappaB transcription factor activity |
| | GO:0051897 | Involved in positive regulation of protein kinase B signaling |

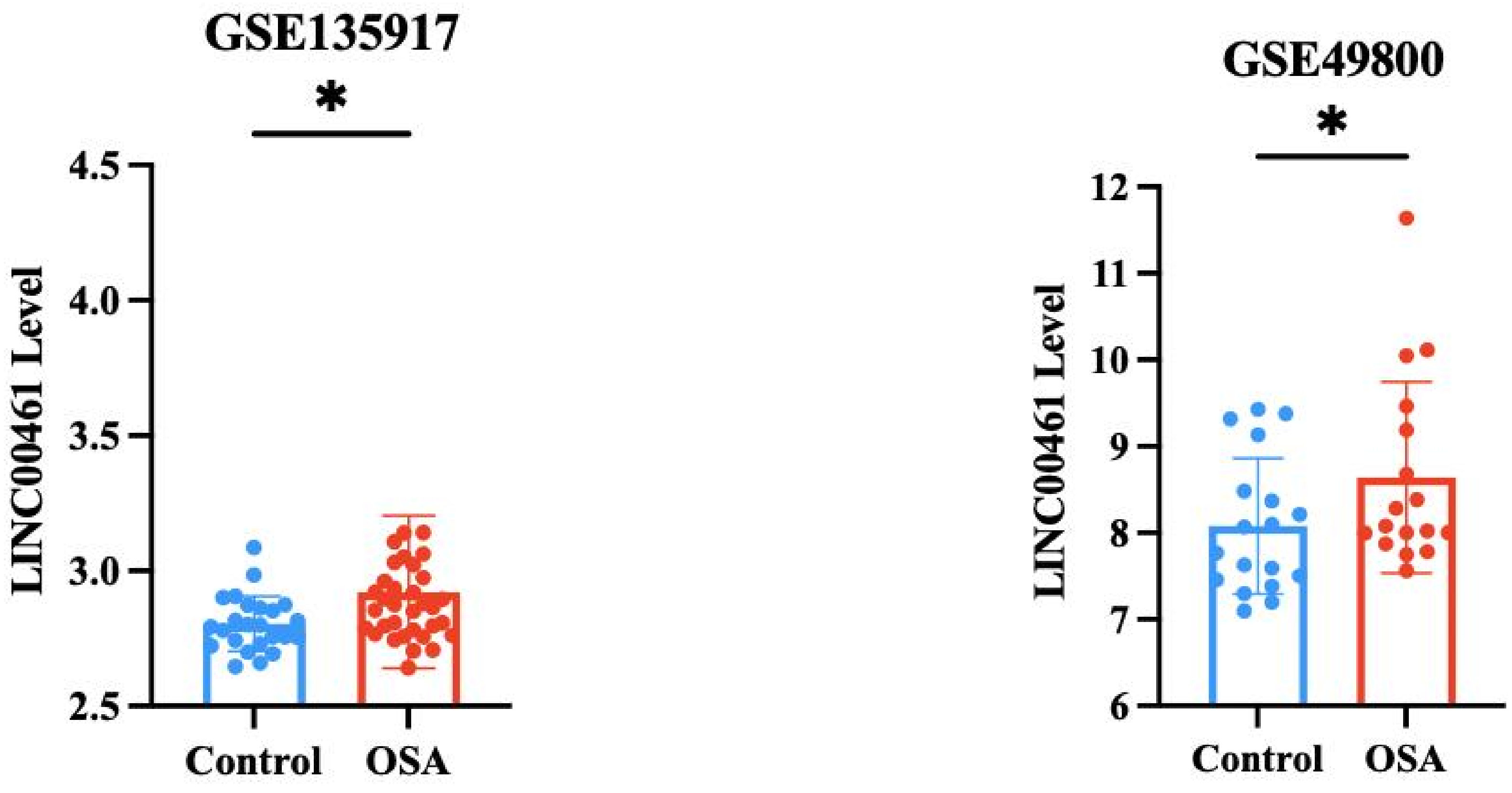


**Figure S2. The upregulation of LINC00461 in OSA patients.** The upregulation of LINC00461 based on two datasets GSE135917 and GSE49800.
